# The neuropsychopharmacology of treatment discontinuation

**DOI:** 10.1038/s41386-026-02518-8

**Published:** 2026-08-10

**Authors:** Baeksun Kim

**Affiliations:** Brain Science Institute, Korea Institute of Science and Technology (KIST), Seoul, Republic of Korea

**Keywords:** Medical research, Neuroscience

## The missing half of the science

**Fig. 1:**
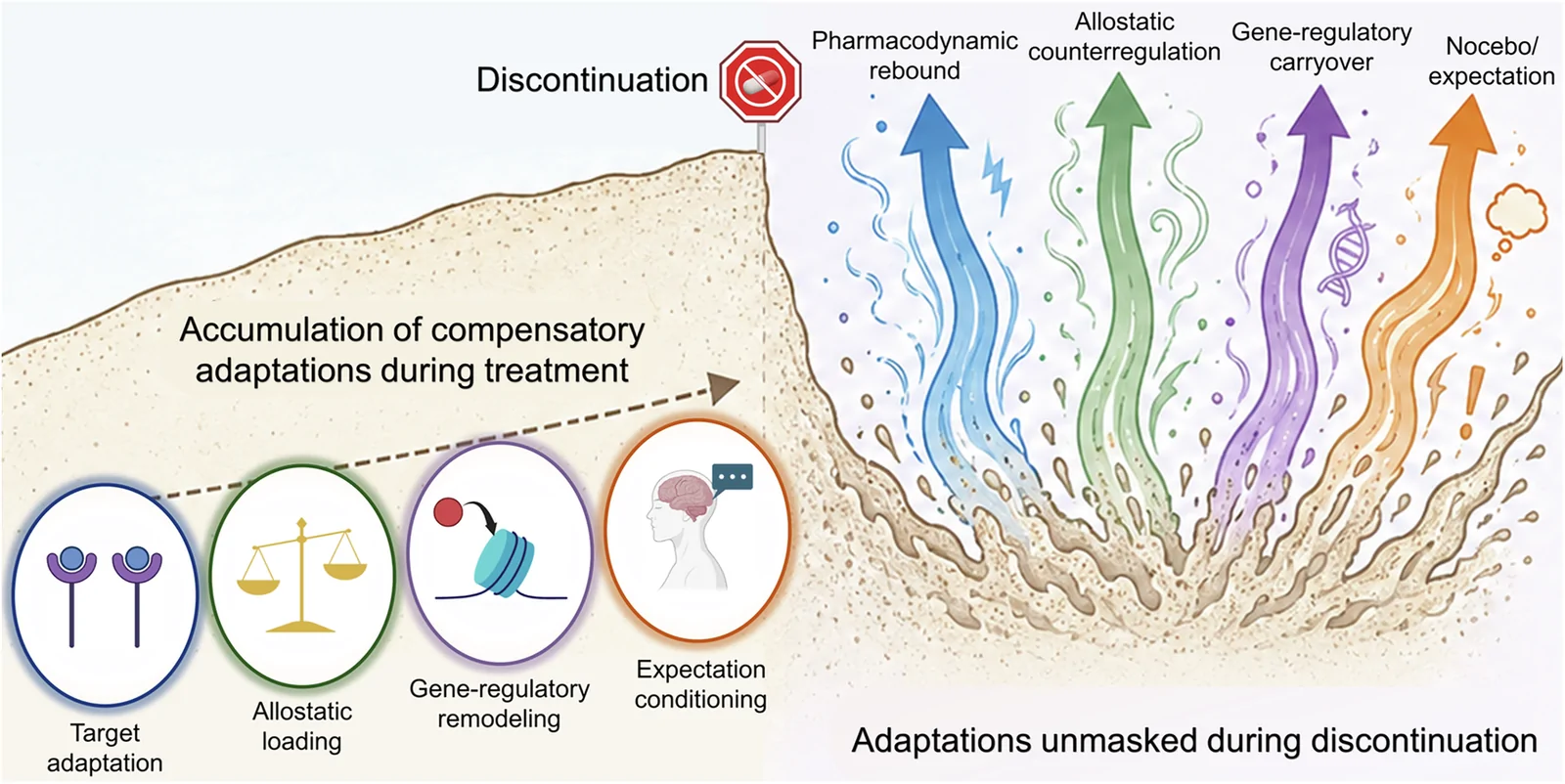
Treatment discontinuation as an active neuroadaptive state. During sustained pharmacotherapy (left), compensatory adaptations accumulate progressively beneath the apparent stability of treatment response. These include target adaptation, allostatic loading, gene-regulatory remodeling, and expectation conditioning. These adaptations are clinically silent during treatment but go unopposed upon drug removal. At discontinuation, the abrupt loss of pharmacological input unmasks all adaptive layers simultaneously, generating an active neuroadaptive state characterized by pharmacodynamic rebound, allostatic counterregulation, gene-regulatory carryover, and nocebo/expectation activation. The trajectory through this discontinuation window—either toward recovery or toward relapse and destabilization—is shaped by the magnitude and interaction of these mechanisms, and by individual differences in vulnerability that current clinical practice does not systematically assess.

Neuropsychopharmacology has spent decades building a detailed science of how a treatment takes hold: receptor occupancy, intracellular signaling, circuit adaptation, and symptom change. What the field has not built, with equivalent rigor, is a science of what happens when the treatment stops. Stopping antidepressants, antipsychotics, benzodiazepines, and other psychoactive drugs is still largely managed through consensus-based tapering and brief monitoring plans. This is pragmatic. It is also a scientific gap masquerading as a clinical solution. Treatment discontinuation is not simply a pharmacological absence. It is an active neuroadaptive state shaped by pharmacodynamic rebound, allostatic counterregulation, and gene-regulatory carryover that can persist well beyond drug clearance (Fig. 1) [1]. The field has a science of treatment effects. Now it needs a science of treatment discontinuation.

## The biology of the “After”

The most immediate consequence of discontinuation is pharmacodynamic. The dose-occupancy relationship is nonlinear and sigmoidal. Therefore, standard dose reductions produce disproportionately large changes in target engagement at the lowest dose range [2, 3]. This is not a logistics problem; it is a mechanistic one that provides the empirical basis for receptor-occupancy-guided tapering [3]. The pharmacodynamic discontinuity can be compounded by receptor-level adaptation. For instance, chronic dopamine D2-receptor blockade induces receptor upregulation that generates behavioral supersensitivity lasting up to several months after drug clearance, surfacing in some patients as withdrawal-emergent psychosis that is easily misread as relapse [4].

These receptor-level events unfold against a background of allostatic counterregulation. On the oppositional-tolerance and allostasis models [5, 6], sustained pharmacotherapy recruits counterregulatory adaptations that directly oppose the drug’s primary effects, potentially implemented through compensatory shifts in excitatory-inhibitory balance and synaptic scaling. When the drug is removed, these forces go unopposed, leaving the brain to settle not at its pretreatment baseline but at a new equilibrium. This framework hypothesizes the post-discontinuation brain as an allostatic product of the treatment, and a critical window of plasticity meriting its own characterization.

A third layer involves gene regulation. Sustained pharmacotherapy may alter transcriptional programs and chromatin-level remodeling mechanisms that shape which genes remain inducible, suppressed, or stress-responsive beyond drug clearance. Such gene-regulatory carryover provides a candidate explanation for a clinically frustrating phenomenon: the patient who responds well, discontinues, and then fails to recapture the same therapeutic response upon re-treatment. If prior treatment has reshaped the gene regulation under which circuits respond to pharmacological input, then re-treatment begins not from the original baseline, but from one altered by prior treatment.

Discontinuation is also not a purely molecular event. Patient expectations about stopping a medication engage nocebo mechanisms in prefrontal-limbic circuits, which are already reorganized by sustained pharmacotherapy. This top-down neurobiological driver interacts with bottom-up pharmacodynamic change in ways that current trial design systematically ignores.

## Clinical lens on discontinuation

Recent meta-analyses agree that treatment discontinuation is a real clinical phenomenon, but disagree on magnitude. Pooled incidence estimates of discontinuation symptoms range sharply from ~15 to ~43% across studies [7–9]. This divergence is expected because discontinuation outcomes are highly heterogeneous, moderated by half-life, duration, taper, expectation, and study design. Now, the question is no longer simply whether discontinuation symptoms occur, but which discontinuation phenotype a given event represents and whether discontinuation reshapes future treatment response.

First, distinguishing pharmacological withdrawal from illness recurrence is central [10]. Withdrawal is marked by onset within days of dose reduction, novel somatic or sensory symptoms not part of the original disorder, a fluctuating course, and rapid resolution on reinstatement; recurrence emerges gradually and reproduces the original phenotype. Yet these features are expert-proposed rather than validated. Separating the phenotypes is therefore an open measurement problem, and a consequential one. Because most trials stop treatment rapidly and rarely measure withdrawal, an event that looks like illness recurrence may in fact be withdrawal-related destabilization [11]. Yet discontinuation can also produce genuine recurrence in some patients, or no recurrence at all in others [12]. Thus, whether a given discontinuation event reflects withdrawal, recurrence, or neither remains unresolved.

Second, prior treatment may alter response to future treatment. This discontinuation-induced refractoriness (the failure to recapture a drug’s original efficacy on re-treatment) is best documented for lithium [13], with suggestive evidence from antidepressants [14], but broader evidence remains unsettled. Therefore, whether discontinuation-induced refractoriness is a general consequence of discontinuation rather than illness progression, and whether it worsens with each successive treat-discontinue cycle, remain important questions for future research.

## A roadmap for the field

Deprescribing is increasingly being recognized as a core psychiatric competency. Therefore, the field demands a scientific substrate across several domains.

Preclinical models must extend beyond 48-h washout windows. Discontinuation studies should be scaled to each drug’s half-life, spanning days to weeks for acute rebound and months for durable adaptations [4, 15]. Studies should compare abrupt cessation, graded tapering, and reinstatement as experimental conditions rather than treating drug removal as a binary event [15]. Because such longitudinal designs multiply time and cost, within-subject monitoring and short-half-life agents would offer lower-burden approaches.

Clinical trials must stop treating discontinuation as an adverse-event appendix. Randomized discontinuation studies should compare taper trajectories rather than durations. They should distinguish withdrawal from recurrence using timing, blinded phenomenological ratings, and reinstatement response. Post-treatment follow-up should make greater use of ecological momentary assessment, actigraphy, sleep architecture, and autonomic indices.

Spanning preclinical and clinical work, predicting individual vulnerability before discontinuation begins is the central unresolved problem. Evidence across drug classes confirms substantial individual variation in discontinuation outcomes [1], yet we currently measure few of the likely determinants before planned discontinuation, including pharmacodynamic adaptation, allostatic burden, or gene-regulatory sensitivity. Identifying who is at risk before discontinuation begins would move the field from managing symptoms to preventing them.

## Conclusion

Discontinuation is an intervention. Like initiation, maintenance, or switching, discontinuation has its own mechanisms, moderators, risks, and therapeutic windows. A field capable of characterizing treatment response with molecular and circuit-level precision should not leave the end of treatment to trial-and-error. Discontinuation is a critical window of plasticity. Studied properly, it will reveal not just why patients relapse and suffer, but how psychiatric treatment can end as thoughtfully as it begins.
